# Improving Neonatal Intensive Care Unit Providers' Perceptions of Palliative Care through a Weekly Case-Based Discussion

**DOI:** 10.1089/pmr.2020.0121

**Published:** 2021-04-16

**Authors:** Jayme D. Allen, Riddhi Shukla, Rebecca Baker, James E. Slaven, Karen Moody

**Affiliations:** ^1^ Department of Pediatrics, Riley Hospital for Children at Indiana University, Indianapolis, Indiana, USA.; ^2^ Indiana University School of Nursing, Indianapolis, Indiana, USA.; ^3^ Department of Biostatistics, Indiana University School of Medicine, Indianapolis, Indiana, USA.; ^4^ Department of Pediatrics, University of Texas MD Anderson Cancer Center, Houston, Texas, USA.

**Keywords:** end-of-life care, interdisciplinary, neonatology, quality of life

## Abstract

***Objective:*** The primary objective was to evaluate the efficacy of a weekly palliative care-guided, case-based discussion of high-risk infants on Neonatal Intensive Care Unit (NICU) physician (MD) and Advanced Practice Provider (APP) perceptions of pediatric palliative care (PPC).

***Study Design:*** The study setting was a level IV academic NICU in a United States midwestern children’s hospital. A pre/post design was used to evaluate the effects of a weekly palliative care-guided, case-based discussion of high-risk infants on neonatology providers’ (MD and APP) perspectives of palliative and end-of-life care in the NICU using a previously published survey instrument. Surveys were completed at baseline and after 12 months of implementation. Data was analyzed with a Wilcoxon Signed Rank test with significance set at *p* < 0.05.

***Results:*** Thirty-one providers (13 APPs and 18 MDs) completed both pre- and post-intervention surveys. Post-intervention, providers were more likely to endorse that they “are comfortable with PPC”, “feel comfortable teaching PPC to trainees”, “feel confident handling end-of-life care”, “have time to discuss PPC”, and “were satisfied with the transition to end-of-life care for their most recent patient”. They also were more likely to report, “families' perception of burden is relevant when making ethical decisions”, that “parents are involved in decisions regarding palliative care”, and that their “institution is supportive of palliative care.” (*p*-values < 0.05 for all).

***Conclusion:*** NICU provider perceptions of palliative care can be improved through the implementation of a case-based interdisciplinary conference that emphasizes palliative care domains in the context of Neonatal ICU care.

## Introduction

Palliative care for infants and children has been defined as “an active and total approach to care, from the point of diagnosis or recognition, throughout the child's life, at the time of death and beyond. It embraces physical, emotional, social, and spiritual elements and focuses on the enhancement of quality of life for the neonatal infant and support for the family. It includes the management of distressing symptoms, the provision of short breaks, and care through death and bereavement.”^1^

National recommendations for incorporating pediatric palliative care (PPC) into the care of newborns at high risk for mortality and morbidity have emerged in order to support quality of life for these babies and their families, guide families in medical decision making, relieve pain and suffering, and provide end-of-life care.^2^ Nonetheless, access to palliative care in the neonatal intensive care unit (NICU) remains limited due to deficits in equipping physicians and nurses with palliative care skills, health care team silos that undermine the development of strong interdisciplinary teams, and insufficient attention to palliative care in medical and nursing schools.^3^

A national survey of neonatologists revealed that there is strong endorsement for palliative care training and also acknowledgment that there remains ongoing difficulty for providers to guide families in the transition from cure-directed, to palliative, and end-of-life care.^4^ A study of bereaved parents and NICU staff reported that end-of-life care practices in the NICU were sometimes perceived as inconsistent among providers.^5^ Areas for improvement in end-of-life care as identified by neonatologists include development of formalized NICU palliative care teams, palliative care provided concurrently with neonatal intensive care, standardization of newborn comfort care guidelines, and institutional programs to reduce clinician compassion fatigue.^1,6^

The aim of this study was to examine the impact of a weekly, NICU case-based discussion, including palliative care and neonatology interdisciplinary teams on NICU providers' perceptions of palliative care using a pre/post study design. NICU providers included neonatologists, neonatology fellows, and advanced practice providers (APPs) in a level IV NICU at a large children's hospital.

## Methods

This study was approved by the Institutional Review Board at Indiana University, which also allowed for a waiver of consent for study participants. NICU and PPC team members identified a mutually agreeable time to hold a weekly interdisciplinary meeting to discuss palliative care domains as they applied to high-risk newborn patients in the NICU. Infants eligible for discussion in the weekly meeting included those admitted to our Level IV academic NICU with significant morbidity and mortality as adapted from guidelines published by Catlin and Carter^7^ shown in Table 1.

**Table 1.** Infant Inclusion Criteria

1	Trisomy 13 or 18
2	Lethal forms of osteogenesis imperfect, inborn errors of metabolism and epidermolysis bullosa
3	Severe hypoxic ischemic encephalopathy
4	Severe CNS malformation (e.g., holoprosencephaly, anencephaly, hydranencephaly)
5	Less than 24-week of gestational age and grade III/IV intraventricular hemorrhage
6	Kidney failure requiring dialysis, and/or liver failure
7	Short gut syndrome with TPN dependence
8	Inoperable congenital cardiac malformations
9	Neurodegenerative disease expected to progress to respiratory failure (e.g., spinal muscular atrophy type 1)
10	Giant omphalocele
11	Congenital diaphragmatic hernia with hypoplastic lungs
12	Any patient NICU team requests to discuss

CNS, central nervous system; NICU, neonatal intensive care unit; TPN, total parenteral nutrition.

Meetings were implemented for one year, and were each attended by 8–16 NICU staff and 2–5 PPC team members in addition to the study physicians. Meetings were one hour long and initially included discussion of four infants, one from each of our four NICU teams. The infants to be discussed were identified by the study team at least 36 hours before the meeting and selection was based on inclusion criteria and discussion with the infant's attending neonatologist. Based on data collected through written program feedback forms, 15 minutes per patient was insufficient to address all palliative care-related domains. Therefore, midway through the year, the number of patients discussed was decreased to two per meeting with two of the four NICU teams contributing patients at each meeting.

The entire NICU staff was notified via secure electronic mail in advance of the meeting regarding which cases would be discussed. The meetings were held in a designated conference room within the NICU space at the same time each week. Before the meetings, the bedside staff involved in caring for the infant were personally invited to these meetings. The meeting was attended by personnel from the PPC team, including any combination of the following team members: PPC physician, APPs, social worker, and chaplain. The NICU team in attendance included neonatologists and APPs caring for the infant, bedside nurse(s), respiratory therapist(s), and support staff including social workers, pharmacists, chaplains, family support coordinators, and child life therapists. A physician member of the research team was also in attendance.

**FIG. 1.**
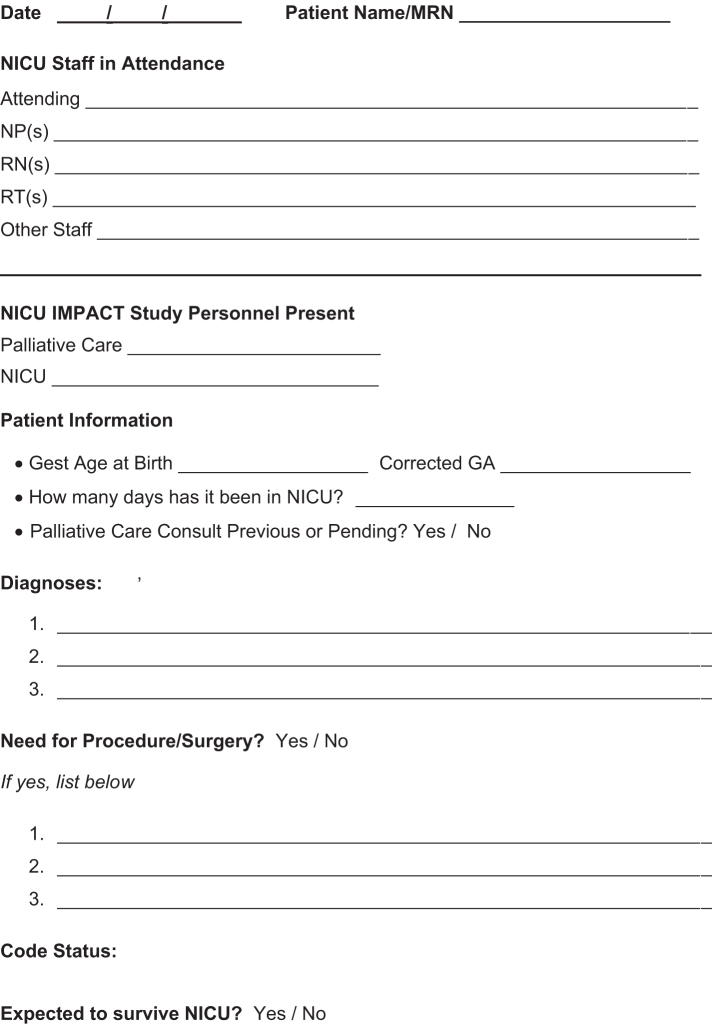

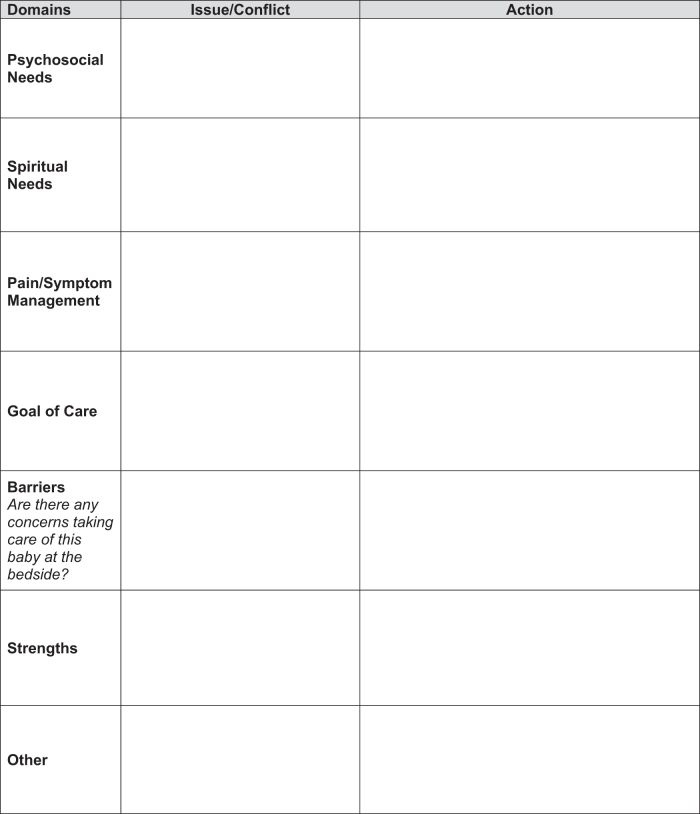
Weekly meeting template form.

The discussion of each case began with an NICU update including the following: a brief overview of the medical course including gestational age, current diagnosis and treatment, code status, anticipated need for surgical intervention, and attending neonatologist prediction of likelihood of survival to discharge. Following the NICU update, a structured discussion using a standardized template was facilitated by the study investigators and guided by the PPC domains of: pain and symptom management, goals of care, spiritual support, and psychosocial aspects including strengths of and challenges for the patient's family (Fig. 1). During discussions, team members from all disciplines were encouraged to express concerns and provide input.

### Outcome measures and data analysis

The primary outcome measure was neonatal providers' perspectives of palliative and end-of-life care in the NICU.^5^ Neonatology staff were surveyed confidentially via electronic mail at baseline and 12 months after implementation of the meetings. This self-administered survey included 28, anchored, 7-point Likert-type scale items (1 = very strongly agree; 2 = strongly agree; 3 = agree; 4 = neutral; 5 = disagree; 6 = strongly disagree; 7 = very strongly disagree) and 5 subsections (demographics and practice characteristics, education, current practices, personal beliefs, and delivery of palliative care) to assess attitudes toward palliative care. It was created through literature review and an expert panel and has been utilized previously in a national survey of neonatologists.^4^

All data were deidentified before analyses by the clinical research coordinator (R.B.).

The secondary outcome was PPC consult rate. Palliative care consultation requests were tracked by the palliative care team as per their standard operating procedures. The total number of NICU admissions was calculated based on physician billing data for the preintervention and post-intervention time periods.

Demographics were reported with raw numbers and descriptive statistics. Pre/post survey data were analyzed using a Wilcoxon signed-rank test; significance was set at *p* < 0.05. Consult rates were compared using chi-square tests for homogeneity. Associations between provider perceptions and provider demographics were assessed with correlation analyses and *t*-tests/analysis of variances depending on if the data were continuous or if the independent variables were categorical. In some instances, categories were collapsed, such as race, where white versus other was used, due to the small numbers of nonwhite participants. All analytic assumptions were verified and all analyses were performed using SAS v9.4 (SAS Institute, Cary, NC).

## Results

Forty-three NICU providers (MD/APP) completed baseline surveys; 31 completed both pre- and 12-month post-intervention surveys. Most providers were female (71%) and white (90.3%). The majority of respondent providers were experienced clinicians with 51.6% having over 10 years of NICU experience. Demographics and practice characteristics of participating NICU providers who completed both pre- and post-intervention surveys are shown in Table 2.

**Table 2.** Demographics and Practice Characteristics (*N* = 31)

Age, mean (SD)	44.13 (13.49)
Gender (%)	
Female	22 (71.0)
Male	9 (29.0)
Race (%)	
Asian	1 (3.2)
Black	1 (3.2)
Unknown	1 (3.2)
White	28 (90.3)
Hispanic	0 (0)
Profession (%)	
Advanced practice provider	13 (41.9)
MD	18 (58.1)
Years of experience in the NICU (%)	
<1	2 (6.5)
1–3	3 (9.7)
4–6	5 (16.1)
7–10	5 (16.1)
>10	16 (51.6)
NICU deaths experienced personally in the past 12 months (%)	
0–3	11 (35.5)
4–7	13 (41.9)
8–11	4 (12.9)
12–15	1 (3.2)
>15	2 (6.5)

Values are mean (SD) for age and frequency (percentage) for all other variables.

SD, standard deviation.

The results of the survey at baseline of neonatal providers' perspectives of palliative and end-of-life care revealed that overall the NICU providers viewed PPC very favorably despite reporting they had little formal education in palliative and end-of-life care. Significant improvements in perceptions from baseline to 12-month follow-up were endorsed by the participants across all the survey subsections, (education, personal beliefs, and palliative care delivery). Particularly encouraging were the improvements in providers' comfort and confidence in delivering PPC. Further analysis by specific provider type (MD vs. APP) was limited by sample size. Survey item scores pre- and post-intervention for the 31 providers completing both time points are shown in Table 3.

**Table 3.** Survey Item Scores Pre- and Post-Intervention (*N* = 31)

Neonatal providers' self-reported perspectives of palliative and end-of-life care survey items (1 = very strongly agree; 2 = strongly agree; 3 = agree; 4 = neutral; 5 = disagree; 6 = strongly disagree; 7 = very strongly disagree)			
Question	Median T1	Median T2	*p*
Education in palliative and end-of-life care			
I received formal education in palliative/end-of-life care	4 (3,6)	5 (3,6)	0.772
I feel comfortable teaching palliative care skills to trainees	**5 (4,5)**	**4 (3,5)**	**0.024**
I feel palliative care is an essential part of training in neonatology	2 (1,3)	2 (1,2)	0.627
Current practices/experiences with palliative care in your NICU			
I have had professional experiences with palliative care	2 (1,3)	2 (1,2)	0.184
I provide families palliative care options	2 (2,3)	2 (1,3)	0.095
I feel comfortable dealing with issues surrounding palliative/end-of-life care	**3 (2,3)**	**2 (2,3)**	**0.002**
I feel confident in my abilities to handle end-of-life care	**3 (2,3)**	**2 (2,3)**	**0.006**
My institution currently does a good job with palliative/end-of-life care	**3 (3,4)**	**2 (1,3)**	**<0.001**
My institution has teams/policies/guidelines to help provide palliative care	**3 (2,4)**	**2 (1,3)**	**0.019**
Parents are involved in decisions regarding palliative care	**2 (1,3)**	**2 (1,3)**	**0.020**
There are adequate services to make referrals for home/inpatient hospice	3.5 (2,4)	3 (2,5)	0.889
Your beliefs about palliative care in your NICU			
There is a place or need for palliative care	1 (1,2)	1 (1,2)	1.000
Families and patients would benefit from palliative care	1 (1,3)	1 (1,2)	0.244
The medical team would benefit from palliative care	2 (1,3)	1 (1,3)	0.564
Palliative care is as important as curative care	2 (1,3)	1 (1,3)	0.348
The families' perception of burden is relevant when making ethical decisions	**1 (1,2)**	**1 (1,2)**	**0.048**
I am satisfied with the transition to end-of-life care for my most recent patient	**3 (2,5)**	**2 (1,3)**	**0.003**
Delivery of palliative care in your NICU			
My institution is supportive of palliative care	**3 (2,3)**	**3 (1,3)**	**0.018**
The physical environment of my NICU is conducive to providing palliative care	**3 (2,4)**	**3 (1,3)**	**0.037**
Palliative care can be emotionally difficult for the team	2 (1,3)	2 (1,3)	0.221
Staff members often disagree around issues of palliative care	3 (2,4)	3 (2,4)	0.326
I view palliative care as a failure of our abilities to take care of a patient	6 (6,7)	6 (6,7)	0.993
It is difficult to determine when to initiate transition to palliative care	3 (3,5)	4 (3,5)	0.249
The uncertainty of a prognosis makes it difficult to provide palliative care	3 (3,4)	4 (3,5)	0.443
There is enough time to have discussions about/give palliative/end-of-life care	**4 (2,5)**	**3 (2,4)**	**0.022**
I have beliefs that at times interfere with my ability to provide palliative/end-of-life care	5 (5,6)	5 (5,6)	0.242
There are adequate places to refer patients when transitioning to palliative care	4 (3,5)	3.5 (3,5)	0.059
There are adequate financial resources to provide palliative care	**4 (4,5)**	**4 (3,4)**	**0.022**

Bold values and superscript significant at p < 0.05.

The palliative care consult rate before beginning the meetings was not statistically different from the post-intervention rate: 33/613 (5.4%) versus 50/737 (6.7%), respectively (*p* = NS). Of note, there were no identified changes in patient acuity, referral patterns, or global practice changes during the study period. There were no significant associations between demographic variables (gender, race, profession, years of experience, and exposure to NICU death) and provider perceptions.

## Discussion

The 2014 Institute of Medicine recommendations include that all health care providers caring for people with advanced illness have at least basic palliative care competencies.^4^

The American Academy of Pediatrics (AAP) Section of Hospice and Palliative Care and Committee on Hospital Care policy statement recommends that all hospitals caring for children with life-threatening illness or those in need of end-of-life care should have a dedicated interdisciplinary specialty palliative care team to support decision making, provide timely and effective interventions to minimize suffering while maximizing quality of life, and manage and coordinate the logistics of care to provide seamless transitions between settings and maintain the highest possible quality of care.^8^ These teams should have sufficient collective expertise to address the physical, psychosocial, emotional, practical, and spiritual needs of the child and family.^5,7^

In an effort to address these recommendations, palliative care education and training courses for neonatology providers have emerged to enhance communication skills for providers^9^ and comfort level for bedside nurses caring for dying infants.^10^ This study is the first to report using a weekly interprofessional case-based discussion to enhance NICU provider comfort and confidence with providing PPC and promote incorporation of palliative care principles into practice.

The weekly meetings conducted during this study allowed for an open discussion among the PPC and NICU interdisciplinary teams regarding management of high-risk newborns within the identified domains of palliative care. This somewhat informal exchange of ideas facilitated the weaving together of palliative care strategies with neonatology standards of practice. This approach allowed the primary neonatology team to integrate palliative care into their routine practice independently as goals of care evolved, referring more complex issues out to the PPC team as needed. As a result, NICU providers felt more comfortable with their own palliative care skills, including their ability to care for infants at end of life and to educate trainees in palliative care. They also felt more supported in terms of time and resources to engage parents in shared decision making, and accordingly, they reported a greater emphasis on the family's value system and parental involvement when making palliative care decisions.

The consultative palliative care model endorsed by the AAP may be ideal, however, due to the widespread need for these services and lack of adequate numbers of training programs for PPC, demand will surely exceed supply for the foreseeable future. Furthermore, there remains the possibility that, in some cases, adding yet another team of providers to a neonate's care could challenge the relationship and primary role of the neonatology team and complicate care further.^11^ The role of the neonatal team as primary throughout the disease course of a neonate mirrors how neonatologists commonly interface with other specialists providing consultation for their patients.^3^

Our intervention did not result in any significant effect on palliative care referral rate. A previously reported four-hour educational initiative in NICU staff reported a significant increase in respondents' reported confidence in making a referral to a tertiary-based PPC team. However, the actual consultation rate before and after the education was not studied.^12^ Our weekly case-based discussions highlighting domains of palliative care may have been predicted to increase the PPC consultation rate due to more familiarity with palliative team members and increased awareness of institutional palliative care resources.

On the contrary, the meetings provided participants with a framework and skills to integrate palliative care into routine NICU care and consequently resulted in greater comfort and confidence with providing palliative care. Therefore, the decision to refer patients for PPC consultation may have been reduced to reflect only those cases requiring a higher level of expertise. It remains unknown if the lack of change in the PPC consultation rate reflects a lack of association or is a result of these two opposing forces.

One limitation of this study is the pre/post design, which did not allow us to control for possible confounding variables such as the general increase in publications regarding the need for palliative care for high-risk neonates, or changes in institutional culture and palliative care services. However, no changes to the structure or availability of palliative care services occurred during the study period. And, although no specific unit-based palliative care education occurred, NICU fellows were provided an annual palliative care communications skills class that was initiated the year before this study period. Another limitation is the decline in post-intervention responses.

Reasons for this decline likely include staff turnover (>12% of MD/APP staff had left the NICU after time 1), lack of meeting participation (only providers with patients to be discussed at the meetings were asked to be present), and work-related factors (workload, time, high patient acuity, etc.). Another possibility and potential limitation is responder bias; nonresponders may have not felt the meetings to be useful as responders. The anonymous nature of the data collection precluded a deeper analysis of these limitations.

In addition, the small sample size did not allow us to determine any demographic predictors of provider perceptions, and the single-institution design limits generalizability of our findings. Study participants were 90% white and 71% female. Therefore, broad applicability of the findings presented here will require study of more gender-diverse and racially diverse provider populations.

In conclusion, NICU physician and APP's perceptions of PPC, including comfort in providing end-of-life care, educating trainees in palliative care, and incorporating families into shared decision making, can be improved through a weekly case-based interdisciplinary conference that emphasizes palliative care domains in the context of NICU care.

## Abbreviations Used

AAP: American Academy of Pediatrics
APP: advanced practice provider
NICU: neonatal intensive care unit
PPC: pediatric palliative care
